# Preliminary Study to Determinate the Effect of the Rearing Managements Applied during Heifers’ Whole Life on Carcass and Flank Steak Quality

**DOI:** 10.3390/foods7100160

**Published:** 2018-10-01

**Authors:** Julien Soulat, Brigitte Picard, Stéphanie Léger, Marie-Pierre Ellies-Oury, Valérie Monteils

**Affiliations:** 1 Université Clermont Auvergne, INRA, VetAgro Sup, UMR Herbivores, F-63122 Saint-Genès-Champanelle, France; julien.soulat@live.fr (J.S.); brigitte.picard@inra.fr (B.P.); marie-pierre.ellies@agro-bordeaux.fr (M.-P.E.-O.); 2 Université de Clermont Auvergne, Université Blaise Pascal, Laboratoire de Mathématiques, BP 10448, F-63000 Clermont-Ferrand, France; Stephanie.Leger@math.univ-bpclermont.fr; 3 CNRS, UMR 6620, Laboratoire de Mathématiques, F-63171 Aubière, France; 4 Bordeaux Science Agro, 1 Cours du Général de Gaulle, CS 40201, F-33175 Gradignan, France

**Keywords:** pre-weaning period, fattening period, growth period, meat sensory properties, rearing managements, rearing surveys

## Abstract

The aim of this study was to investigate the impact of rearing managements applied during a heifers’ whole life on the carcass and flank steak (rectus abdominis) meat traits. For this study, rearing managements applied on 96 heifers were identified by conducting surveys in farms. A heifers’ whole life was divided into three key periods: Pre-weaning, growth, and fattening. The combination of the rearing factors applied during the heifers’ whole life allowed us to characterize several rearing managements. Among them, four have been studied in depth. The main results displayed that the carcass traits were more sensitive to the rearing managements than the flank steak traits. The different managements considered had an impact on the weight, the dressing percentage and the conformation score of the carcass. Whereas, they had no impact on the sensory descriptors, the sheer force and the color of the flank steak. This study showed that the variations observed for carcass and meat traits could not be explained by the variation of only one rearing factor but could be explained by many rearing factors characterizing the rearing management applied. Finally, this study demonstrated that it was possible to improve carcass traits without deteriorating meat traits.

## 1. Introduction

Beef carcass and meat traits are impacted by the animal type (sex and breed) [1,2,3]. Rearing managements have also been shown to influence these characteristics, with the fattening period as the main studied period of the animal’s life. In the literature, the fattening period was mainly studied using experimental devices by controlling one or two rearing factors. The factors that have been most frequently studied during this period are the slaughter age [2,4,5,6], the slaughter weight [7,8], the fattening duration [9,10,11], the fattening diet [12,13,14], and the fattening management, i.e., a pasture period during the fattening period or the whole fattening in housing [15,16,17,18]. The rearing managements applied during the animal’s whole life period (i.e., from birth to slaughter, WLP) are a complex combination of many rearing factors to achieve carcass traits expected by the target market and to maximize their value. Very few studies have jointly studied the influence of the rearing managements applied at different periods of the animal’s life (growth, fattening or whole life) on carcass and meat traits [19,20,21,22]. The aim of the present study was to illustrate the effect of different rearing managements during the animal’s whole life period on carcass traits and meat sensory properties. This study concerned the protected geographical indication (PGI) Fleur d’Aubrac. This PGI was chosen in order to work only on one animal type (i.e., crossbreed Charolais × Aubrac heifers) bred exclusively for the meat production with a slaughter age between 26 and 42 months. In France, the meat consumed mainly comes from female cattle. The shortest life duration of the heifers allows for the improvement of the accuracy of the WLP rearing managements collected by the survey. This study was undertaken using fixed slaughter and post-slaughter conditions to limit the potential bias caused by these parameters on the meat quality [23,24,25].

## 2. Materials and Methods

### 2.1. Animals and Rearing Factors Data Collected by Surveys

The 96 crossbreed Charolais × Aubrac heifers used in this study were born in Occitanie (France region) between December 2012 and May 2013. Their slaughters were distributed between February 2015 and June 2016.

**Figure 1:**
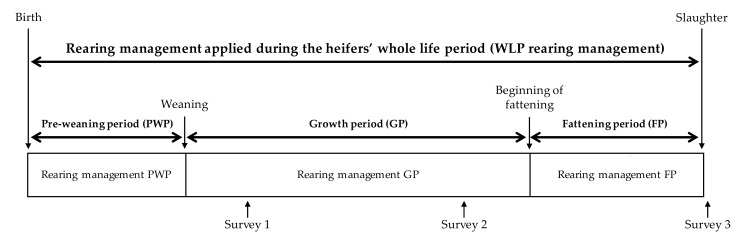
Description of the three key rearing periods during a heifers’ whole life (WLP) and distribution of the three farm surveys conducted over time. Rearing managements characterizing the three key periods (pre-weaning, PWP; growth, GP and fattening, FP) are described in Table 1, Table 2 and Table 3, respectively.

To characterize the WLP rearing management, the heifer’s life was divided into three key periods (Figure 1). Each key period of the heifer’s life was characterized by many quantitative or qualitative rearing factors. The three steps of the survey were distributed over time to allow for the collection of information regarding different rearing factors that were applied during the whole life. The surveys were carried out by interviewing the farmers using questionnaires and establishing batch management practices [26].

In total, 46 rearing factors were used to characterize the three key rearing periods. The rearing factors characterizing the pre-weaning period (PWP, *q* = 16), the growth period (GP, *q* = 13) and the fattening period (FP, *q* = 17) are presented in the Table 1, Table 2 and Table 3, respectively.

The energetic contents of the forages (hays, grass silages and wrapped haylages) in the fattening diet and all concentrates (crude proteins (CP), net energy and neutral detergent fiber (NDF) contents) were recorded. These data were used to calculate the chemical composition of the average forage (i.e., hays + grass silages + wrapped haylages) during the FP and the chemical composition of the average concentrate for each period.

### 2.2. Animals Slaughtering, Carcass Traits and Muscle Sampling

All heifers were slaughtered in the same industrial slaughterhouse (Abattoir du Gévaudan, Antrenas, France). The slaughter was done by exsanguination after stunning under the same conditions for all heifers. Carcasses were suspended vertically using the Achilles method without electrical stimulation. About 1 h (hot carcass weight) post-mortem, carcasses were weighted and graded visually by an official judge (conformation and fat scores) according to the EUROP grid system [27]. Until 24 h post-mortem, carcasses were chilled and stored at 2 °C in a cold room.

The EUROP system consists of a characterization of the carcass conformation using a scoring grid divided into 5 classes: E (extremely muscled), U, R, O and P (very poorly muscled). Each class of conformation was divided into 3 sub-categories using “+” (high), “=” (average), and “−” (low), so that the conformation was divided into 15 subclasses. A scale ranging from 1 (very poorly muscled) to 15 (extremely muscled), corresponding to each of the conformation sub-categories, was considered [27]. In the EUROP system, the fatness score of the carcass is divided into 5 classes where 1 = lean and 5 = very fat. In our study, the fatness score was not a discriminatory trait as all carcasses were scored 3.

Carcass quality was characterized using three measurements: Cold carcass weight (calculated from the hot carcass weight −2%, kg), dressing percentage (dressing% = ratio of cold carcass weight to live weight before slaughter, %) and conformation score. In our dataset, the distribution of each carcass trait was the following: Mean cold weight, 430 kg (standard deviation, SD = 42); mean dressing%, 58.5% (SD = 2.0) and mean conformation score, 11 (U=, SD = 1).

Twenty-four hours post-mortem, the full rectus abdominis (RA) muscle was collected from the right-hand side of the carcass. To conserve the same sampling conditions (24 h post-mortem), irrespective of the slaughtering day, only 77 RA muscles could be taken among the 96 samples. This muscle is an oxidative muscle with higher mean fibre areas [28,29] than the longissimus muscle (LM), which is considered as a reference muscle in most meat quality studies. According to the results of Cassar-Malek et al. [30], the RA muscle is more sensitive to the variation of rearing managements than the LM muscle. Moreover, Oury et al. [31] observed few differences between the sensory properties (tenderness, juiciness and flavor) of both muscles, RA and LM, when heifers were reared under the same conditions.

### 2.3. Meat Quality Evaluation

The color was measured 2 h after excision from the carcass on the fresh RA samples, using a spectrophotometer (Konica Minolta CM-600d, Osaka, Japan) and expressed in CIE L*a*b* units [32]. Before the color measures, meat samples were exposed to air for 20 min. The spectrophotometer was calibrated following the instructions of the manufacturer. A “black” and a “white” calibration were performed before each measurement session. To characterize the color of the whole RA muscle, the mean of 5 measurements (randomly distributed on the muscle) per RA muscle was used. The connective tissues were avoided.

The RA muscles were then weighted, vacuum-packaged and chilled for 14 days at 4 °C for ageing. After ageing, they were frozen at −20 °C until the analysis [33].

Sixteen members of a trained tasting panel conducted the sensory evaluation of the meat, using a monadic test. The members of the tasting panel had attended 20 training sessions before starting the sensory evaluation of the flank steak (RA muscle) samples [34]. In short, the assessors were selected and trained before the final studies in accordance with ISO 8586 [35] and were familiar with sensory assessment of the meat. After the training period of the panel composed of three sessions of different beef samples in which sensory descriptors were defined, assessors rated the descriptors on a structured scale extending from 0 to 10. Then, the concordance between the members of the trained tasting panel was checked using the Kendall concordance test [36].

The samples were randomly selected. Before the tasting session, the meat samples were thawed for 24 h at 4 °C and cut into 2 sub-samples: The first for the sensory evaluation and the second for the sheer force measurement. The sensory evaluation was carried out under conditions in accordance with AFNOR and SSHA [37,38].

The meat samples used for the sensory evaluation were cut into even 15 mm thick steaks. These steaks were cooked on a double-face grill at a temperature of 300 °C for 1 min 45 s in aluminum foil to remove the roasted taste (i.e., up to an internal temperature of 55 °C) [19]. After cooking and removing the meat borders, the steaks were cut into homogeneous pieces (size 15 × 20 × 20 mm) that were served in batches of 3 or 4 on a plastic plate to the trained panel. During each tasting session, 5 samples were evaluated by the trained panel using a Latin square presentation.

The assessors evaluated five sensory descriptors: Initial tenderness, overall tenderness, overall juiciness, flavor intensity and fat presence. Initial tenderness was defined as the tenderness at the first bite, whereas overall tenderness was defined as an evaluation of the tenderness before swallowing the meat sample. Each sensory descriptor was rated on a 10-point non-graduated scale from a score of 0 (tough, dry, slight, and too lean) to a score of 10 (very tender, very juicy, strong, and highly fat).

The sheer force was measured according to the method described by Salé [39,40] on raw meat using material testing equipment (MTS Synergie 200). For each sample, 25 meat portions with different thicknesses (max 18 mm) were cut perpendicular to the fibers [41]. From the different measurements obtained, the force (daN) and the work (dJ) at 10 mm were determined. The sheer force was then calculated (ratio of work to force, dJ/daN).

In our dataset, the distribution of each flank steak trait was the following: Mean weight, 1.6 kg (SD = 0.2); mean L*, 26.2 (SD = 3.2); mean a*, 15.1 (SD = 2.5); mean b*, 10.9 (SD = 2.9); mean initial tenderness, 6.3/10 (SD = 0.8); mean overall tenderness, 6.1 (SD = 0.8); mean overall juiciness, 6.4 (SD = 0.5); mean flavor intensity, 4.5 (SD = 0.7); mean fat presence, 7.6 (SD = 0.6) and mean sheer force, 0.5 dJ/daN (SD = 0.07).

### 2.4. Statistical Analyses

The statistical analyses were performed using R 3.2.3 [42].

A descriptive analysis of the dataset to observe the normality of distribution was realized, using quantile-quantile plots [43]. After this descriptive analysis, the quantitative rearing factors that were discontinuous with few values were converted into qualitative parameters. The transformed rearing factors concerned: (i) For the growth period: The calculated average percentage of each forage (hay, grass silage, and wrapped haylage) in the housing diet and the growth period duration (Table 2); (ii) for the fattening period: The calculated average of the concentrate’s crude protein content across the whole period (Table 3). These conversions allowed for the definition of homogeneous modality classes that could be processed statistically.

From the rearing factors characterizing the pre-weaning period (*q* = 16, Table 1), PWP rearing management clusters (PWP-clust) were constructed using a factor analysis for mixed data (FAMD), followed by a hierarchical clustering on the principal components (HCPC). The FAMD allowed for the consideration of quantitative and qualitative rearing factors simultaneously. The number of rearing management clusters was determined from the obtained dendrograms. For the growth and fattening periods, the same procedure was applied from the rearing factors characterizing these both periods (GP-clust and FP-clust), *q* = 13 (Table 2) and *q* = 17 (Table 3), respectively. The three FAMD were realized from the rearing data of the 96 heifers. The FAMD and HCPC were implemented using the “FactoMineR” package [44] in R.

Between the rearing management clusters defined for each key period of the heifer’s life, analysis of variance (ANOVA) or χ^2^ tests were conducted for each of the quantitative or qualitative rearing factors to evaluate their dependence on the defined rearing management clusters. If there was a significant difference in ANOVA, a Tukey test was performed to compare the average pairwise, using the “agricolae” package in R [45].

The WLP rearing management was determined from the combination of the obtained rearing management clusters (PWP-clust × GP-clust × FP-clust) in which the heifers were affected. Only the WLP rearing managements with at least 8 heifers for carcass and meat data were considered in the following study.

For each of the carcass and meat traits, an ANOVA was realized to evaluate their dependence on the considered WLP rearing managements. In the ANOVA, the effect of the farm was tested on all carcass and meat traits. If the effect of the farm was significant, the farm was considered as a random effect for the analysis. For each sensory parameter, the score given by each member of the trained tasting panel was used in the analysis. The effects of the member of the trained tasting panel and the effect of the animal were tested in the ANOVA. If they were significant, these factors were considered as random effects.

Finally, to investigate the effects of the WLP rearing managements on the carcass and meat traits, if there was not random effect, ANOVAs were performed. If not, mixed models were developed using the “lmerTest” package [46] in R. If the results of the ANOVA or mixed models were significant, a post-hoc Tukey test was conducted to compare the average pairwise. For the mixed models, the Tukey test was realized using the package “multcomp” [47] in R.

The effects were declared significant at *p* ≤ 0.05 and tending toward significant was considered for 0.05 < *p* ≤ 0.10.

## 3. Results

After the HCPC analysis, three different rearing management clusters were defined for each key period of the heifer’s life (PWP, GP and FP).

### 3.1. Description of the Three Rearing Management Clusters Obtained for the Pre-Weaning Period (PWP-Clust)

The PWP-clust1 contained 37 calves. The properties of this PWP rearing management cluster were an average daily gain significantly lower than the both other PWP clusters and the longest pasture duration (Table 1). The concentrate’s crude protein and energy contents were lower than the other rearing managements applied during the pre-weaning period. During the housing, the time spent by the calf with its mother was longer than both of the other rearing managements applied during the pre-weaning period, as the calves were permanently with their mothers. Finally, in PWP-clust1, calves did not have concentrates during the pasture period.

The PWP-clust2 contained 39 calves. This PWP rearing management cluster was characterized by a shorter pasture duration than both other PWP rearing managements (Table 1). In this cluster, the concentrate’s crude protein and energy contents had intermediate values compared to both of the other rearing managements applied during the pre-weaning period. In the PWP-clust2, the calves ingested concentrates only during the housing period of the pre-weaning period. Finally, in this rearing management, 66.7% of the calves were born without the intervention of the farmer.

The PWP-clust3 contained 20 calves. The properties of this cluster were a longer period with concentrate in the calves’ diet and an intermediate pasture duration than the other rearing managements applied during the pre-weaning period. During the pasture, the calves received concentrate (Table 1). Moreover, in PWP-clust3, the concentrate’s crude protein and energy contents were significantly higher than both of the other rearing managements applied during the pre-weaning period. The average daily gain of the calves from PWP-clust2 and PWP-clust3 was higher than that of the calves from PWP-clust1. In the PWP-clust2 and PWP-clust3, the time spent by the calf with her mother during the housing was the lowest. The calves were with their mother only during feeds.

### 3.2. Description of the Three Rearing Management Clusters Obtained for the Growth Period (GP-Clust)

The GP-clust1 contained 21 heifers. This GP rearing management cluster was characterized by the lowest weaning weight and the longest pasture duration (Table 2). In the GP-clust1 and GP-clust2, the period during which the heifers received concentrates in their diet was shorter than that in GP-clust3. In GP-clust1; the concentrate’s crude protein content was lower compared to the other rearing managements applied during the growth period. During the housing period, the heifers received hay (between 20% and 40%) and grass silage (>50%). This management was applied for more than 500 days. In the GP-clust1, 95.2% of the heifers received a hay complement during the whole pasture duration of the growth period.

The GP-clust2 contained 46 heifers. This GP rearing management cluster had the shortest pasture duration (Table 2). In the GP-clust1 and GP-clust2, the heifers ingested a lower concentrate quantity during the growth period than those in the GP-clust3. Among the heifers in the GP-clust2, 82.6% ingested mostly hay (>80% or between 40 and 80% in diet) and 32.6% ingested also wrapped haylage (between 40 and 60% of the diet), during the housing period of the growth period. Then, 87% of the heifers had no grass silage in their diet. In the GP-clust2, the heifers were not supplemented with hay during the pasture of the growth period.

The GP-clust3 contained 29 heifers. This GP rearing management cluster was characterized by the longest period during which heifers received concentrate in their diet and the highest concentrate quantity intake (Table 2). In this cluster, the concentrate’s crude protein and energy content values were the highest. This rearing management was applied for more than 500 days and during pasture of the growth period, the heifers were not supplemented with hay.

### 3.3. Description of the Three Rearing Management Clusters Obtained for the Fattening Period (FP-Clust)

The FP-clust1 contained 20 heifers. Properties of this FP rearing management cluster were a longer fattening period and an older age at slaughter than the other rearing managements applied during the fattening period (Table 3). In the FP-clust1, the heifers were younger and lighter at the beginning of the fattening period than those from the other fattening rearing managements. In the FP-clust1 and FP-clust2, the heifers’ average daily weight gain was higher than that in the FP-clust3. In FP-clust1, the fattening diet offered contained on overage 36.9% hay, 36.5% wrapped haylage and 20.4% grass silage. Then, the forage’s crude protein and NDF contents had intermediate values and the concentrate’s energy content had lower values than those obtained in the other fattening rearing management clusters. In the FP-clust1, 78.1% of the heifers received a mean concentrate with more than 40 g/kg dry matter (DM) of crude proteins during the whole of the fattening period. In the FP-clust1 and FP-clust2, heifers had higher concentrate quantities during the whole of the fattening period than those from the FP-clust3. In FP-clust1, the heifers’ fattening was carried out in housing.

The FP-clust2 contained 21 heifers. This rearing management cluster was characterized by heifers which had a higher initial weight and were slaughtered older than those from the FP-clust1 (Table 3). In this rearing management, the fattening diet was composed mostly of hay (87.8%) and the proportions of grass silage and wrapping haylage were the lowest. In the FP-clust2, the forage’s crude protein and NDF content values were lower and higher respectively than those obtained in the other fattening rearing managements. Then, the crude protein content of the mean concentrate given, for the whole rearing period was below 250 g/kg DM. In FP-clust2, the main fattening managements were housing (34.9%) and outside (34.9%).

The FP-clust3 contained 49 heifers. The properties of this rearing management cluster were a lower average daily gain than FP-clust1 and the heifers were slaughtered with the lowest weight (Table 3). In this fattening rearing management, the highest proportion of grass silage (52.7%) and the lowest proportion of hay (18.3%) compounded the fattening diet. Then, the heifers ingested the lowest concentrate quantities and had the longest pasture duration. In the FP-clust3, the forage’s crude protein and NDF content values were higher and lower respectively than those obtained in the other rearing management FP clusters. Then, the crude protein content of the mean concentrate given, for the whole fattening period was below 250 g/kg DM. In the FP-clust2 and FP-clust3, the fattening duration was shorter than that in the FP-clust1. In the FP-clust3, the main fattening managements were pasture (52.4%) and pasture and housing (42.8%).

### 3.4. Description of the WLP Rearing Managements Considered in This Study

From the rearing management (PWP, GP and FP) clusters, 12 different WLP rearing managements characterizing the whole life of the heifers are defined (Table 4). These rearing managements were the combination of rearing management clusters applied during the three key phases (pre-weaning, growth and fattening) of the heifers’ life. For the rest of the study, only four WLP rearing managements: WLP-A, WLP-D, WLP-E, and WLP-F were considered to have at least eight heifers for the carcass and the meat data. The heifers from the WLP-A management received the rearing management clusters as following: PWP-clust1, GP-clust1, and FP-chust3, during their life. During the WLP-D management, the rearing management clusters applied during the heifers’ life were: PWP-clust1, GP-clust3, and FP-clust2. The rearing management clusters characterizing the WLP-E were as following: PWP-clust2, GP-clust2, and FP-clust1. Finally, the rearing management cluster: PWP-clust2, GP-clust2, and FP-clust2 characterized the rearing management applied during the heifers’ life, WLP-F.

The WLP-A and WLP-D had only the pre-weaning rearing management in common. The WLP-E and WLP-F received the same rearing managements during the pre-weaning and growth period. WLP-D and WLP-F had only the fattening rearing management in common. The WLP-A and WLP-D were mostly characterized by a lower average daily weight gain than WLP-E and WLP-F during the pre-weaning period. Then, during the growth and fattening periods, the heifers ingested a higher quantity of concentrate in the WLP-A than WLP-D (Table 2 and Table 3). In the WLP-E and WLP-F, the heifers ingested higher concentrate quantities than WLP-A only during the fattening period. In WLP-E, the fattening period duration was the longest and the heifers were slaughtered the youngest. During the fattening period, the heifers ingesting the highest hay percentage and the lowest grass silage and wrapping haylage percentage received the WLP-D and WLP-F managements. The WLP-A was characterized by the longest pasture duration during the heifers’ life and the lightest heifers at slaughter. The heifers were slaughtered at the same age in the WLP-A, WLP-D and WLP-F managements.

### 3.5. Impact of the Four *WLP* Rearing Managements on Carcass and Flank Steak Traits

The WLP-E and WLP-F allowed for the production of a heifers’ carcass that was heavier and had a higher conformation score than WLP-A (Table 5). The WLP-D, WLP-E, and WLP-F managements produced heifers’ carcasses with similar weights and conformation scores.

These difference in carcass traits, between these WLP rearing managements, could be explained by the distribution of the different conformation classes according to the EUROP grid. The conformation score of the carcasses from WLP-A was mainly U = (42%) but over half of the carcasses (52.6%) were conformed to U− and R+. In contrast, no carcasses with an R+ conformation were obtained and only one with a U− conformation score when heifers received the WLP-E. With the WLP-E, the conformation scores of carcasses produced by heifers were mainly U = (68.4%) or higher (26.3% of U+ and E− conformation scores). The heifers receiving the WLP-F produced carcasses with U+ and U= conformation scores (46.1% and 30.8%, respectively). The WLP-D allowed for the production of carcasses with a higher dressing% than WLP-A.

The four rearing managements applied during the heifers’ whole life had no significant impact on the flank steak traits in terms of weight, all sensory descriptors, sheer force and color (Table 6).

## 4. Discussion

### 4.1. Carcass Traits

The carcass results of this study match those of Hennessy et al. and Greenwood et al. [48,49] illustrating that animals with faster growth during the pre-weaning period were slaughtered with heavier carcass weights when the growth and fattening managements were similar. For the same slaughter weight, Cerdeño et al. [50] did not show significant differences on the conformation score and dressing% of calves when different rearing managements were applied during the pre-weaning period. However, if the calves were slaughtered higher, the conformation score and the dressing% of their carcass were improved [51]. The rearing management applied during the pre-weaning period could have an impact on the carcass traits. During the growth period, Guerrero et al. [52] did not observe any significant impact of rearing managements (intensive vs. extensive) on carcass traits (carcass weight, dressing% and conformation score) in young bulls with the same fattening management. The absence of significant differences in carcass traits between WLP-D and WLP-F is in accordance with these results. The effect of the rearing managements applied before weaning or fattening could interact with those applied during the next heifers’ life period involving an attenuation or an amplification of the effects on carcass traits.

During the fattening period, the heifers from WLP-E were slaughtered at a younger age than those receiving the other rearing managements applied during the heifers’ whole life. In the literature, the fattening period was the most frequently studied period and many rearing factors were known to influence carcass traits. Our results disagree with the results of many studies, which displayed that the heifers slaughtered at an older age were heavier [2,5]. The heavier carcass from WLP-E could be explained by the fattening period duration. This result agrees with many studies indicating an increase in the carcass weight of cull cows with an extension of the fattening period duration [10,11,53]. However, the WLP-D and WLP-F, with a shorter fattening period duration, obtained a similar carcass weight than WLP-E. In accordance with Soulat et al. [54], this result showed that is possible to attain the same carcass traits with different rearing managements applied during the fattening period. In our study, the heifers with the highest concentrate intake during the fattening period produced heavier carcasses confirming the results observed by Cook et al. [55] in heifers. However, our results displayed that it is possible to obtain the same carcass weight from a rearing management during the heifers’ life using lower concentrate quantities in the diet during the growth and fattening periods. For the same concentrate quantity intake, in our study, the forage composition of the fattening diet had no impact on the carcass weight. This result is in accordance with many studies which did not find evidence for an impact of the fattening diet composition on carcass weight, in young bulls, steers, and cull cows [14,56,57]. In WLP-A, the heifers had the longest pasture duration during their life. This management produced lighter carcasses than the other rearing managements considered in this study. In accordance with these results, Keane and Allen and Neel et al. [15,58] observed a lighter carcass in steers from the fattening managements involving pasture. The longest pasture period of heifers during their whole life could explain part of the difference observed on the carcass weight between our four rearing managements applied during the heifers’ whole.

Although the slaughter ages were different between WLP-E and WLP-D, the dressing% of the carcasses were not significantly different. This result is in accordance with the results of Bures and Barton [2] in heifers. Few studies observed the effect of this rearing factor on the dressing% in heifers. Many studies observed a higher dressing% when young bulls were slaughtered older [6,59]. However, our results showed that the slaughter age was not the only rearing factor that could have an impact on the dressing%. For a similar slaughter age, we observed a difference in the dressing% between carcasses from WLP-A and WLP-D. According to our results, a longer fattening period duration could have no impact on the dressing%. This result disagrees with the results of many studies indicating an increase in the dressing% of dairy cull cows when the fattening duration was longer [10,60]. The animal type and the breed, which were different could explain this difference. These parameters are known to influence the dressing%, which was higher for carcasses from WLP-D than WLP-A. In WLP-A, the heifers were slaughtered at a lighter weight. This result confirms the results of McEwen et al. [7] who observed an increase in the dressing% when steers were slaughtered at a heavier weight. However, our results showed that the dressing% was not systematically increased when heifers were slaughtered heavier. Moreover, these data illustrate that the variation observed for dressing% was not explained only by the live weight of the heifers. In contrast to the results of Price et al. [61] in young bulls and steers, in our study, the combination of a higher hay percentage in the fattening diet and heavier slaughter weight did not lead to a lower dressing%. As demonstrated in many studies, the fattening diet composition could have no impact on the dressing% in cattle [55,57,62,63]. It could explain the few differences observed between the four rearing managements applied during the heifers’ whole life considered in our study. In the WLP-A, the heifers had the longest pasture duration during their life and their carcasses had a lower dressing%. In accordance with the results of Keane and Allen and Neel et al. [15,58], the pasture duration could explain in part the low dressing% values observed.

In our study, the heifers slaughtered younger had a similar conformation score than those slaughtered older. This result complies with the studies of Bures and Barton and Ahnstrom et al. [2,5]. However, with a similar slaughter age, the carcasses from the WLP-A had a lower conformation score than the carcasses from WLP-F. As for the others carcass traits, the variation of the conformation score could not be explained by only one rearing factor [54]. In accordance with different results obtained in cull cows [10,11,53], WLP-E with the longest fattening period allowed to produce carcasses with higher conformation scores. However, our results showed that it is possible to obtain conformation scores similar to WLP-E with a shorter fattening period. In our study, the conformation score did not seem to be impacted by the slaughter weight. This observation confirms the results obtained by Keane and Allen and Ellies-Oury et al. [15,64] in steers. According to many studies, the fattening diet composition would not seem to have any impact on the conformation score in heifers and steers [55,61,63]. On the other hand, WLP-A with the longest pasture duration produced carcasses with the lowest conformation score (R+). The pasture duration during the heifers’ whole life could explain a part of the variation of the conformation score observed between our four rearing managements applied during the heifers’ whole life. This observation confirms the results observed by Keane and Allen and Neel et al. [15,58] during the fattening period, in steers. However, the conformation scores were similar between WLP-A and WLP-D. Our study displayed that the rearing management applied during the heifers’ whole life and not only one rearing factor and one heifers’ life period explained the carcass traits variability. This study showed that an improvement of the carcass quality traits could be obtained from different rearing managements during the life of heifers. Moreover, our results showed that it is possible to modify the rearing managements at different period of heifers’ life without having a negative impact on carcass traits.

### 4.2. Meat Traits

In accordance with the results of Hennessy and Morris [48], the variations in the growth rate during the pre-weaning period observed between the rearing managements considered in this study did not seem to have any impact on the meat’s sensory traits. In the study conducted by Hennessy and Morris [48], steers and heifers were subjected to the same rearing managements after weaning in contrast to our study. Our results also confirm those of Picard et al. [65] showing that modifications of the rearing managements during the pre-weaning period had no impact on the fiber properties measured at slaughter on 18-month-old bulls However, Cerdeño et al. [50] showed that the rearing management applied during the pre-weaning period can have an impact on the tenderness of the calf meat. Considering the rearing management applied during the heifers’ whole life, it is possible that the effect on the sensory properties of the flank steak could interact with the rearing managements applied after the weaning period. To the best of our knowledge, the impact of the rearing managements during the pre-weaning period on sheer force and meat color of the meat has never been studied before.

Our results also match the results of Durunna et al. [66] obtained on LM in steers. They showed that modifications of the rearing managements during the growth period did not seem to have an impact on sensory properties or on meat color. It is possible that the rearing managements during the fattening period could mitigate a possible negative impact on the meat, resulting from the rearing managements applied during the previous periods (pre-weaning and growth) of the heifers’ life. Thanks to the plasticity of the muscle properties, this could explain the absence of differences between the rearing managements considered in our study with regard to meat traits.

In contrast to our results, Oury et al. [19] observed an impact of rearing managements applied during the heifers’ whole life on flank steak tenderness. In cull cows, Couvreur et al. [20] also observed an impact of the rearing managements applied during the fattening period on the tenderness and juiciness of the flank steak. The different rearing managements and animal types (sex, breed, age) could explain the differences between these studies and our results. In the study of Oury et al. [19], the heifers ingested maize silage and higher concentrates quantities during the fattening period compared to our study. Other studies have demonstrated an effect of the animal type and breed on the LM meat traits in cattle [1,2,67]. Furthermore, Ellies-Oury et al. [64] demonstrated that an increase in the slaughter weight tended to increase tenderness and flavor intensity scores of flank steak in steers. In our study, the slaughter weight had no impact on the sensory properties of the flank steak. Moreover, as we worked at the scale of the combination of many rearing managements applied at different periods of the animal’s life, it is difficult to identify the individual impact of the slaughter weight on the meat traits.

With regard to the RA color, Serrano et al. [68] did not demonstrate any effect of the fattening diet in young bulls. In their study, Oury et al. [69] observed few impacts of the rearing managements applied during the heifers’ whole life on the L* parameter for flank steak. Our results are in accordance with these studies. However, there are few data on the impact of rearing factors on RA color in cattle. In our study, the different rearing managements considered allowed us to attain the same meat color.

Our study also showed that different combinations of rearing managements during the heifers’ life could allow us to attain the same, or to improve the carcass and meat traits. That confirmed our previous results obtained for carcass and LM meat traits [54,70,71].

In our study, we observed that the carcass quality was weakly related to the meat quality of RA. Carcasses characterized by a low weight, conformation score, and dressing% can produce the same flank steak quality compared to carcasses which had high values for these three parameters. This result is in accordance with the observation of Gagaoua et al. [72] showing that a low contribution of carcass traits can explain meat traits of a cull cow during the fattening period. For other muscles, Bonny et al. [73] observed a very weak relationship between the carcass traits (conformation and fatness scores) and the sensory meat properties. However, a recent study showed that the carcass traits (fatness score, percentages of muscle and fat in the carcass) can have an impact on the sensory properties (tenderness, flavor and juiciness) of the meat [74]. The rearing management applied during the heifers’ whole could improve carcass traits without decline RA meat traits.

A limitation of this study was the low number of animals for certain groups. To confirm the robustness and the repeatability of this approach it would be important to increase the number of heifers per rearing management. To complete this study, it would be interesting to expand to other rearing managements applied during the heifers’ life such as those based on maize silage or corn. It would also be interesting to study other muscles to evaluate whether the effects of the rearing managements applied during the heifers’ whole life are similar irrespective of the considered muscle. Finally, a consideration of the production costs and carcass valorization per rearing management would help meat sector stakeholders to reduce costs without declining the carcass traits and the eating quality of meat.

## 5. Conclusions

To conclude, the originality of this study was to consider the rearing managements applied during the heifers’ whole life and to study their effects on carcass and meat traits. Our results showed that the rearing managements applied during the heifers’ whole life seemed to have more impact on carcass traits than on the flank steak properties. According to the rearing managements considered in our study, the carcass traits could be improved without altering the meat properties. Different combinations of rearing managements during a heifers’ life have been identified to improve the production at the farm level.

## Figures and Tables

**Table 1:** Description of the rearing factors characterizing the pre-weaning period (PWP) and the PWP rearing management clusters obtained for this period (PWP-clust).

Rearing Factors	Description of the Modalities	Overall (*n* = 96)			PWP Rearing Management Clusters									*p*
					PWP-Clust1 (*n* = 37)			PWP-Clust2 (*n* = 39)			PWP-Clust3 (*n* = 20)			
**Quantitative Rearing Factors**		**Mean**	**SD**	**SE**	**Mean**	**SD**	**SE**	**Mean**	**SD**	**SE**	**Mean**	**SD**	**SE**	
Birth weight (kg)	Calf weight at birth	42	4	0.4	42	4	0.7	41	4	0.6	41	5	1.1	0.80
ADG_PWP (kg/day)	Average daily gain of the calf during PWP	1.0	0.1	1.0	0.9 ^b^	0.2	0.03	1.1 ^a^	0.1	0.01	1.0 ^a^	0.1	0.02	<0.001
Age of the cow (years)	Age of the heifer’s mother at the heifer’s mother	6.9	2.9	0.3	7.5	3.2	0.5	6.0	2.3	0.37	7.5	3.0	0.7	0.04
Age at the first calving (months)	Age of the heifer’s mother at her first calving	34.5	3.0	0.3	33.7	3.3	0.5	34.9	2.8	0.4	35.2	2.4	0.4	0.09
Duration_day_CC (hour/day)	Time spent per day by the calf with her mother during the housing period	11.9	11.3	1.1	22.8 ^a^	1.6	0.3	4.1 ^b^	8.6	1.4	7.2 ^b^	10.4	2.3	<0.001
Tot_duration_CC (days)	Total time spent by the calf with her mother between the birth and the weaning	192.0	67.8	6.9	252.6 ^a^	38.2	6.3	144.3 ^b^	44	7.0	173.0 ^b^	64.5	14.4	<0.001
Conc_duration_PWP (days)	Number of days of offered concentrates in the diet during PWP	81.4	61.5	6.3	53.6 ^b^	36.0	5.9	71.3 ^b^	41.6	6.7	152.4 ^a^	77.5	17.3	<0.001
Conc_CP_PWP (g/kg DM)	Calculated average of the concentrate’s crude proteins content across the whole PWP	100	55	5.6	64 ^c^	41	6.7	93 ^b^	18	2.9	178 ^a^	46	10.3	<0.001
Conc_E_PWP (Mcal/kg DM)	Calculated average of the concentrate’s energy content across the whole PWP	0.8	0.5	0.05	0.5 ^c^	0.3	0.05	0.8 ^b^	0.2	0.03	1.5 ^a^	0.3	0.07	<0.001
Pasture_duration_PWP (days)	Number of days spend on pasture during PWP	140.3	23.6	2.4	163.0 ^a^	14.7	2.4	122.0 ^c^	11.2	1.8	133.9 ^b^	19.5	4.4	<0.001
PWP_duration (days)	Duration of PWP	253.9	34.9	3.6	257.6	39.0	6.4	251.3	28.4	4.5	252.1	39.4	8.8	0.72
**Qualitative Rearing Factors**														
Insemination type														0.11
Artificial	Artificial insemination using frozen semen	53.1%			45.9%			61.5%			50%			
Natural	Insemination realized by a bull	46.9%			54.1%			38.5%			50%			
Calving														<0.001
Easy	Natural calving	78.1%			86.5%			66.7%			85%			
Help	Farmer intervention during calving	21.9%			13.5%			33.3%			15%			
Bull type														0.004
Bull-3 years	3-year-old bulls belonging to the farmer	34.4%			35.1%			41.0%			20%			
Bull->3 years	Bull older than 3 years belonging to the farmer	27.1%			21.6%			30.8%			30%			
Bull-IA-CE	Artificial insemination from frozen semen for calving ease	12.5%			10.8%			12.8%			15%			
Bull-IA-EM	Artificial insemination from frozen semen for early muscularity	11.4%			10.9%			7.7%			20%			
Bull-IA-CE & EM	Artificial insemination from frozen semen for calving ease and early muscularity	14.6%			21.6%			7.7%			15%			
Conc_housing_PWP														<0.001
Yes	Offered concentrates in housing calve diet during PWP	88.5%			81.1%			100%			80%			
No	No offered concentrates in housing calve diet during PWP	11.5%			18.9%			0%			20%			
Conc_pasture_PWP														<0.001
Yes	Offered concentrates in pasture during PWP	20.8%			0%			0%			100%			
No	No offered concentrates in pasture during PWP	79.2%			100%			100%			0%			

DM: dry matter. *n*: number of heifers. SD: standard deviation. SE: standard error. Values followed by different letters (a–c) are significantly different from each other at *p* ≤ 0.05.

**Table 2:** Description of the rearing factors characterizing the growth period (GP) and the GP rearing management clusters obtained for this period (GP-clust).

Rearing Factors	Description of the Modalities				GP Rearing Management Clusters									*p*
		Overall (*n* = 96)			GP-Clust1 (*n* = 21)			GP-Clust2 (*n* = 46)			GP-Clust3 (*n* = 29)			
**Quantitative Rearing Factors**		**Mean**	**SD**	**SE**	**Mean**	**SD**	**SE**	**Mean**	**SD**	**SE**	**Mean**	**SD**	**SE**	
Age at weaning (months)	Age of heifer at the weaning	8.5	1.1	0.1	8.5	1.4	0.3	8.5	0.7	0.1	8.3	1.3	0.2	0.75
Weaning weight (kg)	Heifer weight at the weaning	296	47	4.8	260 ^b^	53	11.6	311 ^a^	29	4.3	299 ^a^	52	9.7	<0.001
ADG_GP (kg/day)	Average daily gain of the heifer during GP	0.6	0.1	0.01	0.6	0.1	0.02	0.5	0.1	0.01	0.7	0.1	0.01	0.60
Conc_duration_GP (days)	Number of days of offered concentrates in the diet during the GP	206.4	118.1	12.0	133.0 ^b^	0.0	0.0	150.9 ^b^	80.4	11.8	347.6 ^a^	84.7	15.7	<0.001
Conc_quanti_GP (kg)	Total concentrate quantity intake during GP	293.4	249.5	25.5	166.3 ^b^	0.0	0.0	140.0 ^b^	95.2	14.0	628.7 ^a^	190.5	35.4	<0.001
Conc_CP_GP (g/kg DM)	Calculated average of the concentrate’s crude proteins content across the whole GP	99	59	6.0	44 ^c^	0.0	0.0	95 ^b^	56	8.3	145 ^a^	48	8.9	<0.001
Conc_E_GP (Mcal/kg DM)	Calculated average of the concentrate’s energy content across the whole GP	0.9	0.5	0.05	0.5 ^b^	0.0	0.0	0.6 ^b^	0.3	0.04	1.5 ^a^	0.4	0.07	<0.001
Pasture_duration_GP (days)	Number of days spend on pasture during GP	272.5	51.1	5.2	349.0 ^a^	0.0	0.0	241.3 ^c^	24.8	3.6	266.4 ^b^	33.2	6.2	<0.001
**Qualitative Rearing Factors**														
Hay_GP (%)	Across the whole GP, the calculated average percentage of hay in the housing diet													<0.001
>80%		23.9%			0%			50%			0%			
(40%; 80%)		21.9%			0%			32.6%			20.7%			
(20%; 40%)		37.5%			100%			17.4%			24.1%			
<20%		16.7%			0%			0%			55.2%			
Grass_silage_GP (%)	Across the whole GP, the calculated average percentage of grass silage in the housing diet													<0.001
>50%		29.2%			100%			0%			24.1%			
<50%		15.6%			0%			13.0%			31.0%			
0%		55.2%			0%			87.0%			44.9%			
Wrapped_haylage_GP (%)	Across the whole GP, the calculated average percentage of wrapped haylage in the housing diet													<0.001
>60%		15.6%			0%			4.4%			44.8%			
(40%; 60%)		15.6%			0%			32.6%			0%			
<40%		16.7%			0%			13.0%			34.5%			
0%		52.1%			100%			50%			20.7%			
GP_duration (days)	The duration of GP													<0.001
>500 days		76.0%			100%			50%			100%			
<500 days		24.0%			0%			50%			0%			
Nature of pasture														<0.001
Grass	During above 75% of the pasture period, the heifer diet was only grass	79.2%			4.8%			100%			100%			
Grass & Hay	During above 75% of the pasture period, the heifer diet was grass and a hay complement	20.8%			95.2%			0%			0%			

DM: dry matter. *n*: number of heifers. SD: standard deviation. SE: standard error. Values followed by different letters (a–c) are significantly different from each other at *p* ≤ 0.05.

**Table 3:** Description of the rearing factors characterizing the fattening period (FP) and the FP rearing management clusters for this period (FP-clust).

Rearing Factors	Description of the Modalities				FP Rearing Management Clusters									*p*
		Overall (*n* = 96)			FP-Clust1 (*n* = 20)			FP-Clust2 (*n* = 21)			FP-Clust3 (*n* = 43)			
**Quantitative rearing factors**		**Mean**	**SD**	**SE**	**Mean**	**SD**	**SE**	**Mean**	**SD**	**SE**	**Mean**	**SD**	**SE**	
Age early fattening (months)	Age of the heifer at the beginning of FP	26.9	2.6	0.2	23.9 ^b^	2.1	0.5	28.2 ^a^	0.8	0.2	28.7 ^a^	1.4	0.06	<0.001
Slaughter age (months)	Age of the heifer at the slaughter	33.0	3.0	0.3	30.7 ^b^	2.4	0.5	34.5 ^a^	2.9	0.6	33.3 ^a^	2.0	0.3	<0.001
Initial weight (kg)	Live weight of the heifer at the beginning of FP	607	56	5.7	585 ^b^	66	14.7	621 ^a^	47	10.2	613 ^ab^	46	7.0	0.02
Slaughter weight (kg)	Live weight of the heifer before the slaughter	734	62	6.3	745 ^a^	41	9.2	747 ^a^	68	14.8	691 ^b^	58	8.8	<0.001
ADG_FP (kg/day)	Average daily gain of the heifer during FP	0.7	0.3	0.03	0.8 ^a^	0.3	0.07	0.7 ^ab^	0.4	0.02	0.5 ^b^	0.2	0.03	0.01
Hay_FP (%)	Calculation of the hay percentage in the average diet across the whole FP	55.6	34.3	3.5	36.9 ^b^	23.7	5.3	87.8 ^a^	14.9	3.2	18.3 ^c^	0.8	0.1	<0.001
Grass_silage_FP (%)	Calculation of the grass silage percentage in the average diet across the whole FP	22.5	23.0	2.3	20.4 ^b^	12.9	2.9	9.3 ^c^	13.2	2.9	52.7 ^a^	23.6	3.6	<0.001
Wrapping_haylage_FP (%)	Calculation of the wrapped haylage percentage in the average diet across the whole FP	18.5	23.4	2.4	36.5 ^a^	23.6	5.3	0.7 ^b^	4.6	1.0	27.4 ^a^	20.3	3.1	<0.001
Forage_CP_FP (g/kg DM)	Calculated average of the forage’s crude proteins content across the whole FP	106	31	3.2	108 ^b^	5	1.1	88 ^c^	36	7.8	139 ^a^	5	0.8	<0.001
Forage_E_FP (Mcal/kg DM)	Calculated average of the forage’s energy content across the whole FP	1.2	0.1	0.01	1.2	0.1	0.02	1.2	0.1	0.02	1.2	0.1	0.6	0.10
Forage_NDF_FP (g/kg DM)	Calculated average of the forage’s NDF content across the whole FP	577.0	43.5	4.4	579.5 ^b^	28.7	6.4	606.6 ^a^	26.1	5.7	512.5 ^c^	1.5	0.2	<0.001
Conc_quanti_FP (kg)	Total concentrate quantity intake during FP	823.1	486	49.6	954.4 ^a^	539.9	134.4	970.2 ^a^	383.1	85.6	321.6 ^b^	153.2	23.4	<0.001
Conc_E_FP (Mcal/kg DM)	Calculated average of the concentrate’s energy content across the whole FP	1.9	0.1	0.01	1.9 ^b^	0.1	0.02	1.9 ^a^	0.04	0.01	1.9 ^a^	0.001	0.0001	<0.001
Pasture_duration_FP (days)	Number of days spend on pasture during FP	50.4	74.0	7.5	0.0 ^c^	0.0	0.0	57.3 ^b^	88.5	19.3	113.3 ^a^	34.6	5.3	<0.001
FP_duration (days)	Duration of FP	207.4	125.9	12.8	273.8 ^a^	168.9	37.8	192.3 ^b^	84.8	18.5	137.0 ^b^	57.6	8.8	<0.001
**Qualitative rearing factors**														
Conc_CP_FP (g/kg DM)														<0.001
>250 g/kg DM	Across the FP, the calculated average of the concentrate’s crude proteins content was above 250 g/kg DM	26.0%			78.1%			0%			0%			
<250 g/kg DM	Across the FP, the calculated average of the concentrate’s crude proteins content was below 250 g/kg DM	74.0%			21.9%			100%			100%			
Fattening management														<0.001
Pasture	The fattening was carried out on pasture	15.6%			0%			9.3%			52.4%			
Outside	The fattening was carried outside without grass	16.7%			0%			34.9%			4.8%			
Pasture & Housing	The fattening was started at pasture and then finished in housing	18.7%			0%			20.9%			42.8%			
Housing	The fattening was carried out in housing	49.0%			100%			34.9%			0%			

DM: dry matter. *n*: number of heifers. SD: standard deviation. SE: standard error. Values followed by different letters (a–c) are significantly different from each other at *p* ≤ 0.05.

**Table 4:** Description of the rearing managements applied during the heifers’ whole life period (WLP).

WLP Rearing Managements	Rearing Management Clusters			Number of Heifers	
	PWP	GP	FP	Carcass Data	Meat Data
WLP-A	PWP-clust1	GP-clust1	FP-clust3	19	18
WLP-B	PWP-clust1	GP-clust2	FP-clust2	1	1
WLP-C	PWP-clust1	GP-clust3	FP-clust1	6	6
WLP-D	PWP-clust1	GP-clust3	FP-clust2	11	8
WLP-E	PWP-clust2	GP-clust2	FP-clust1	19	12
WLP-F	PWP-clust2	GP-clust2	FP-clust2	13	10
WLP-G	PWP-clust2	GP-clust3	FP-clust1	1	1
WLP-H	PWP-clust2	GP-clust3	FP-clust2	6	4
WLP-I	PWP-clust3	GP-clust1	FP-clust3	2	2
WLP-J	PWP-clust3	GP-clust2	FP-clust1	6	6
WLP-K	PWP-clust3	GP-clust2	FP-clust2	7	6
WLP-L	PWP-clust3	GP-clust3	FP-clust2	5	3

The WLP rearing managements were obtained from the rearing management clusters. The rearing management clusters were defined for each key period of heifers’ life (pre-weaning, PWP; growth, GP and fattening, FP), described in Table 1, Table 2 and Table 3.

**Table 5:** Impact of the four rearing managements applied during the heifers’ whole life period (WLP) on carcass traits.

Carcass Traits	WLP Rearing Managements ^1^												*p*
	WLP-A (*n* = 19)			WLP-D (*n* = 11)			WLP-E (*n* = 19)			WLP-F (*n* = 13)			
	Mean	SD	SE	Mean	SD	SE	Mean	SD	SE	Mean	SD	SE	
Carcass weight (kg)	393 ^b^	36	8.3	422 ^ab^	46	13.9	435 ^a^	21	4.8	446 ^a^	43	11.9	<0.001
Dressing (%)	57.3 ^b^	1.6	0.4	59.1 ^a^	2.2	0.7	58.6 ^ab^	1.3	0.3	58.5 ^ab^	1.3	0.4	0.01
Conformation score (scale 1–15)	10.3 ^b^	0.9	0.2	10.8 ^ab^	0.9	0.3	11.3 ^a^	0.6	0.1	11.2 ^a^	0.8	0.1	0.002
Number of carcasses per EUROP classes ^2^ (proportion of each conformation score)													
E−	0 (0%)			0 (0%)			1 (5%)			0 (0%)			
U+	1 (5%)			3 (27%)			4 (22%)			6 (46%)			
U=	8 (41%)			3 (27%)			13 (68%)			4 (31%)			
U−	6 (32%)			5 (46%)			1 (5%)			3 (23%)			
R+	4 (22%)			0 (0%)			0 (0%)			0 (0%)			

*n*: number of heifers. SD: standard deviation. SE: standard error. Values followed by different letters (a,b) are significantly different from each other at *p* ≤ 0.05. ^1^, The WLP rearing management was the combinations of the rearing managements applied during the pre-weaning (PWP), growth (GP) and fattening (FP) periods of the heifers. The WLP rearing management A was the following combinations of PWP-clust1, GP-clust1 and FP-clust3. The WLP rearing management D was the following combinations of PWP-clust1, GP-clust3 and FP-clust2. The WLP rearing management E was the following combinations of PWP-clust2, GP-clust2 and FP-clust1. The WLP rearing management F was the following combinations of PWP-clust2, GP-clust2 and FP-clust2. The traits of the rearing management clusters are presented in the Table 1, Table 2 and Table 3. ^2^, The EUROP classes are E+ (extremely muscled), E=, […], P= and P− (very poorly muscled). In our dataset, conformation score of carcasses was only between E− and R+.

**Table 6:** Impact of the four rearing managements applied during the heifers’ whole life period (WLP) on flank steak (rectus abdominis, RA) traits.

Meat Traits	WLP Rearing Managements ^1^												*p*
	WLP-A (*n* = 18)			WLP-D (*n* = 8)			WLP-E (*n* = 12)			WLP-F (*n* = 10)			
	Mean	SD	SE	Mean	SD	SE	Mean	SD	SE	Mean	SD	SE	
RA weight (kg)	1.4	0.2	0.04	1.5	0.2	0.07	1.6	0.2	0.06	1.7	0.2	0.06	0.35
Sensory descriptors of RA muscle (scale 0–10) ^2^													
Initial tenderness	6.4	1.9	0.4	6.2	1.9	0.7	6.2	1.7	0.5	6.2	1.9	0.5	0.90
Overall tenderness	6.2	2.0	0.5	6.0	1.9	0.7	5.9	1.8	0.5	5.9	1.9	0.5	0.89
Overall juiciness	6.5	1.7	0.4	6.4	1.6	0.6	6.6	1.7	0.5	6.7	1.7	0.5	0.47
Flavour intensity	4.6	2.3	0.5	4.7	2.4	0.8	4.5	2.3	0.7	4.3	2.2	0.7	0.48
Fat presence	7.5	2.3	0.5	7.8	2.3	0.8	7.5	2.4	0.7	7.5	2.2	0.7	0.68
Sheer force (dJ/daN)	0.49	0.06	0.01	0.53	0.08	0.03	0.48	0.07	0.02	0.53	0.09	0.03	0.24
Colour													
L*	26.3	2.5	0.6	27.9	3.4	1.8	26.8	2.7	0.8	25.1	2.9	0.9	0.20
a*	15.0	2.4	0.6	13.9	2.0	0.7	15.4	2.1	0.6	14.4	3.1	1.0	0.50
b*	11.6	3.1	0.7	10.2	2.0	0.7	10.3	1.6	0.5	10.7	3.9	1.2	0.52

*n*: number of heifers. SD: standard deviation. SE: standard error. ^1^, The WLP rearing management was the combinations of the rearing managements applied during the pre-weaning (PWP), growth (GP) and fattening (FP) periods of the heifers. The WLP rearing management A was the following combinations of PWP-clust1, GP-clust1 and FP-clust3. The WLP rearing management D was the following combinations of PWP-clust1, GP-clust3 and FP-clust2. The WLP rearing management E was the following combinations of PWP-clust2, GP-clust2 and FP-clust1. The WLP rearing management F was the following combinations of PWP-clust2, GP-clust2 and FP-clust2. The traits of the rearing management clusters are presented in the Table 1, Table 2 and Table 3. ^2^, Scale for initial tenderness, overall tenderness, overall juiciness, flavour intensity, fat presence and overall appreciation: 0 = tough, dry, slight, too lean and highly disliked and 10 = very tender, very juicy, strong, highly fat and highly liked.
